# Hearing Aid apps: are they safe, practical and beneficial for children and teens in challenging situations?

**DOI:** 10.1007/s00405-024-08851-2

**Published:** 2024-07-31

**Authors:** Dzemal Gazibegovic, Andrea Bohnert, Anne Katrin Laessig

**Affiliations:** 1 Audiology Division, Department of Otolaryngology, Head and Neck Surgery, University of Mainz, Langenbeckstrasse 1, 55131 Mainz, Germany; 2 Communication Disorders Division, Department of Otolaryngology, Head and Neck Surgery, University of Mainz, Langenbeckstrasse 1, 55131 Mainz, Germany

**Keywords:** Hearing aids, App, Children, Teenagers, Noise reduction, Beam Forming

## Abstract

**Purpose:**

An adult version of an app giving users the control over the level of the volume, microphone directionality and noise reduction was adapted for children. The main purpose of this study was to evaluate the effect of changes made to microphone directionality and noise reduction in the myPhonak Junior (the app) on Speech intelligibility in challenging listening environments in children and teens.

**Methods:**

The randomized, non-blinded interventional study with a single group of subjects involved two study visits with a home trial in-between. In the final study session speech assessment in noise was conducted in three different, randomly assigned conditions: default mode (Autosense Sky OS), preffered (self-adjusted) and the extreme condition. Questionnaire based assessment was conducted to assess the subjective benefit of using the app in different daily situations.

**Results:**

The best scores (speech results in noise) were achieved with the preferred setting and the default Autosense Sky OS setting was significantly better than the extreme setting. The self-reported benefit through the questionnaire indicates significantly better result when adjusting the hearing aids through the app.

**Conclusion:**

The app is an easy-to-use way of controlling the level of noise reduction and the beam forming for children 11 years and older. It has the potential to help customizing the hearing aids beyond the default setting and helping to improve speech understanding in noise.

## Introduction

Speech recognition and understanding in noisy environments is always a particular challenge for people with hearing loss, but it is especially difficult for children and adolescents [1, 2]. This is particularly problematic for school age children in noisy, reverberant classrooms when sitting at a distance from the teacher. Directional microphones and noise reduction features in hearing aids (HAs) can improve the signal to noise ratio and lead to improved speech recognition [3]. However, these technologies offer limited benefits in certain situations e.g. when the speaker is behind [4, 5].

Recent research does not provide a consistent view on the benefits of directional microphones in all situations. Wolfe [4] observed a small decrement in speech perception with an adaptive directional microphone setting when the speaker was behind. However, the benefit when the speaker was in front exceeded this. Thus, they concluded that the directional setting was better than the omnidirectional setting overall. In a later study, Wolfe [5] found no effect on speech perception of noise reduction and the adaptive directional microphone combined, regardless of signal direction. But the children preferred to use the noise reduction and directional settings. Gustafson [6] also found that an adaptive directional microphone improved speech perception for some children but increased difficulty for others. When speech was from behind, listening effort increased and speech perception reduced. Conversely, Browning [2] reported that the directional microphone provided benefit regardless of the target location.

This previous research highlights that one setting may not be suitable for all children in all situations [5]. However, the difficulties of asking children to make frequent changes to their hearing aid settings cannot be underestimated. They are unlikely to manually switch consistently to the microphone setting that optimizes the signal to noise ratio [7]. This issue is partly solved with modern HAs with automatic switching systems (e.g. AutoSense Sky OS in Phonak). These control the switching between omnidirectional and directional microphones autonomously and adjust the level of noise suppression based on scene analyses.

Directional microphones can be useful in many different acoustic environments such as at home, at school, or in social situations. There are also situations in which directional microphones may not be beneficial or may even be detrimental e.g. when speech is not from the front [4]. This is likely to be true both for automatic systems where the HA itself makes the selection and where the child is expected to make the decision. Wolfe [8] showed that children expressed a strong preference for noise reduction technologies when speech was from the front and back. However, other studies have shown that listener preference and the automatic switching algorithm were not always in agreement [7]. Thus, improved benefits for listening in noise with directional microphones and noise reduction features may be achievable if children were able to easily adjust a limited number of settings themselves.

Phonak has adapted the adult myPhonak app specifically for children and created a “myPhonak Junior” (the app) that gives users the control over the level of the volume, microphone directionality (Beam Forming-BF) and noise reduction (NR) in their hearing aids (HAs) [9]. This provides an alternative approach to using the settings selected automatically by the AutoSense Sky OS, based on scene analysis. It allows users to make the beamformer narrower or wider and the noise reduction weaker or stronger based on preferences in specific listening environments.

### Study objectives

The primary objective was to evaluate the effect of changes made to microphone directionality (Beam Forming-BF) and noise reduction (NR) in the myPhonak Junior (the app) on Speech intelligibility in challenging listening environments in children and teens (ages 8–17).

The preferred setting with BF and NR adjusted individually was compared with the automatically activated speech in noise program in AutoSense Sky OS 3.0 (NR, BF) at the default volume.

The secondary objectives were to gain knowledge regarding:

- Any potential negative impact on speech understanding in noise with the extreme settings for NR and BF, with BF set to minimum, NR set to maximum.
- The child’s ability to understand how the sliders in the app work and how they impact the sound of the hearing aid.
- The child’s perception of benefits of having these features accessible through the app following the home trial.

We hypothesize that the adjustments made by the child in noisy situations with the defined and limited feature set in the app leads to an equal or increased hearing performance in noise situations compared to the hearing performance of the child in the automatic mode.

### Subjects

**Fig. 1:**
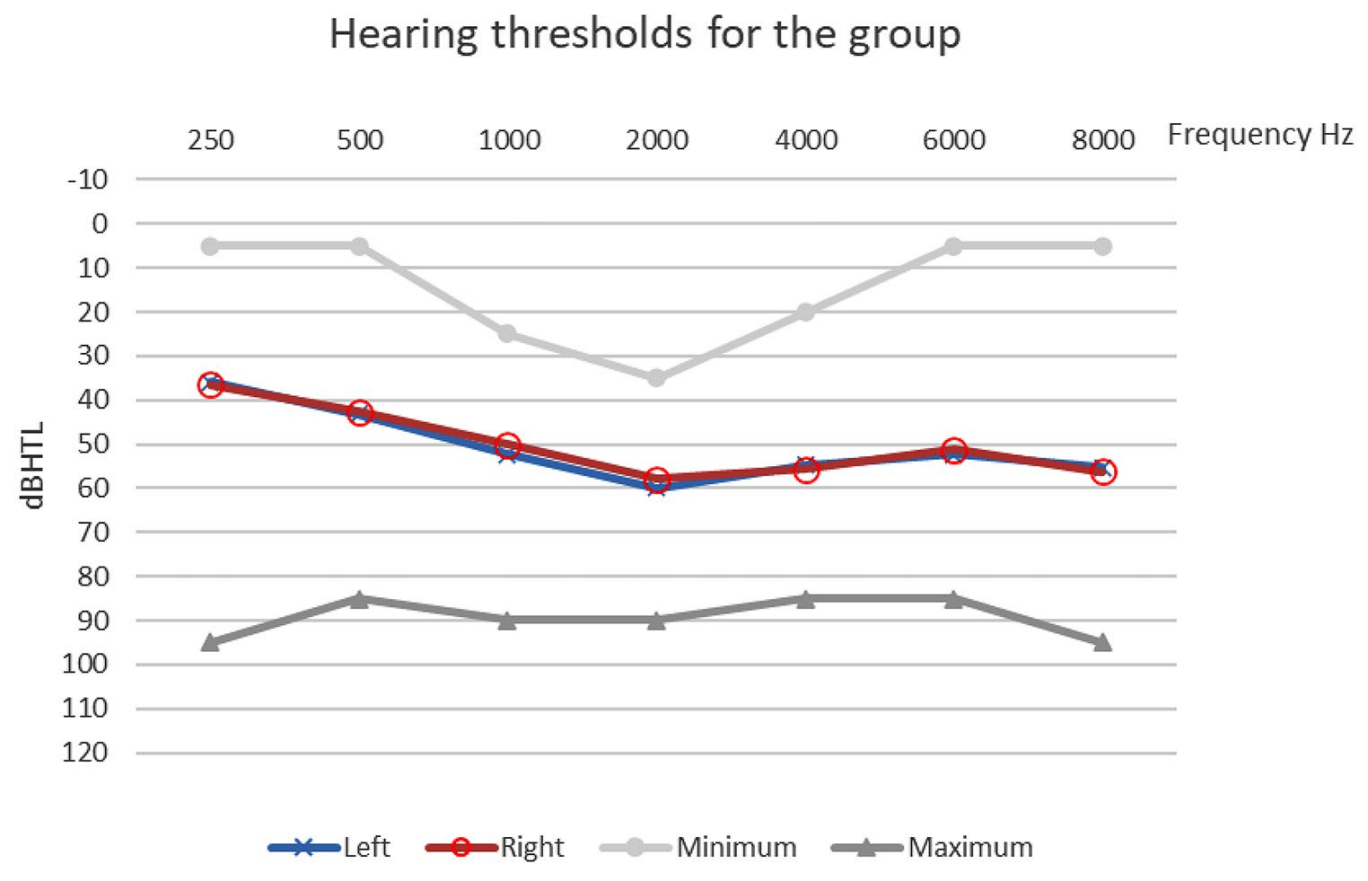
Range of hearing loss in subjects: The red and blue curve indicates the average per side and the grey the maximum and minimum Hearing loss for the entire study group

Sixteen children aged between 8 and 17 years were invited to participate in the study. Inclusion criteria were that they had a bilateral, mild to severe hearing loss and be experienced users of the Phonak Sky HA for at least 6 months. All children fulfilling the inclusion criteria who came for the regular clinical check-up over a period of one year were invited to participate in the study. Exclusion criteria were any additional disabilities to the hearing impairment or inability to complete the study procedures. Mean age was 12.3 (SD 2.55 ) years with a median and range of 12 (8–17) years. The range and mean hearing loss of the 16 subjects is shown in Fig. 1.

Parents confirmed agreement for their child to participate in the study by signing the informed consent. Ethics committee approval was gained from the “Landesärztekammer des Landes Rheinland-Pfalz” and the clinical trial registry number was 2020–15,352.

A full data set was available for 15 subjects. One child dropped out after the first study session due to technical issues with the app.

## Method

The study was a randomized, non-blinded interventional study design, using a single group of subjects.

At the start of the study the fitting and adjustments from subjects’ own HAs were transferred 1:1 to a study version of either Sky M90-M or Sky M90-SP HA. Coupler measurements were carried out to ensure identical transfer of the fittings from own to the study HA. Also, each child confirmed that the sound perception and quality with the study devices did not differ from their own HAs. The use of the dedicated study devices was necessary to ensure compatibility with the app. Furthermore the study HAs were paired with the app installed on the child’s/family’s phone or a loaner phone.

Study subjects attended two in-clinic sessions with a home trial period of a minimum of two weeks in between.

During the first visit the myPhonak Junior app was demonstrated and discussed. A Practical Hearing Aids Skill Test checklist (PHAST) was used as a basis to demonstrate and check in a structured manner the children’s understanding and ability to adjust the HA through the app. The PHAST was carried out in a clinic office with a simulated speech in noise environment.

### Test conditions

Speech intelligibility testing was then carried out in three conditions: Autosense Sky OS, preferred and extreme. For the preferred setting, sentences were presented continuously at a level of 70dB SPL with cafeteria noise at 65dB SPL, and each child was asked to adjust the amount of noise reduction using the Noise Reduction slider (NR) &/or the beamforming width using the Speech Focus (SF) sliders in the app from the default position to an alternate position to create a “Preferred” setting. The slider settings for the preferred condition was noted and children were not allowed to adjust it further during the test. The noise reduction slider adjusts the noise reduction from Off (0) to Strong (10). Speech Focus sets the BF to most open at “0” and to the narrowest at “10”. For the extreme setting the NR was at 10, and BF was 0. In the Autosense Sky OS condition, with 65dB of noise, AutoSense Sky OS will classify the environment as speech in noise, and will set the default noise reduction slider to 3 and the Speech Focus slider will default to 8.

### Speech Intelligibility testing

Speech intelligibility of sentences in noise was tested using the adaptive Oldenburg Sentence Test (OLSA) [10]. To ensure the hearing aids have classified the environment as speech in noise, the cafeteria Noise with speech babble was played for a minute before testing with a constant level of 65dB SPL and the child was instructed to sit comfortably facing the centre speaker. With the cafeteria noise played constantly a list of 20 sentences was presented from the centre speaker with an adaptive loudness so that a signal to noise ratio for a speech reception threshold (SRT) of 50% was calculated. The order of test conditions was randomized. Prior the study all children had previous experience with a version of the adaptive speech in noise testing, with either the OLSA or OLKISA (version for younger children) already. However, the specific adaptation of the test with the usual speech shaped noise being replaced by the Cafeteria Noise was new to all participants.

**Fig. 2:**
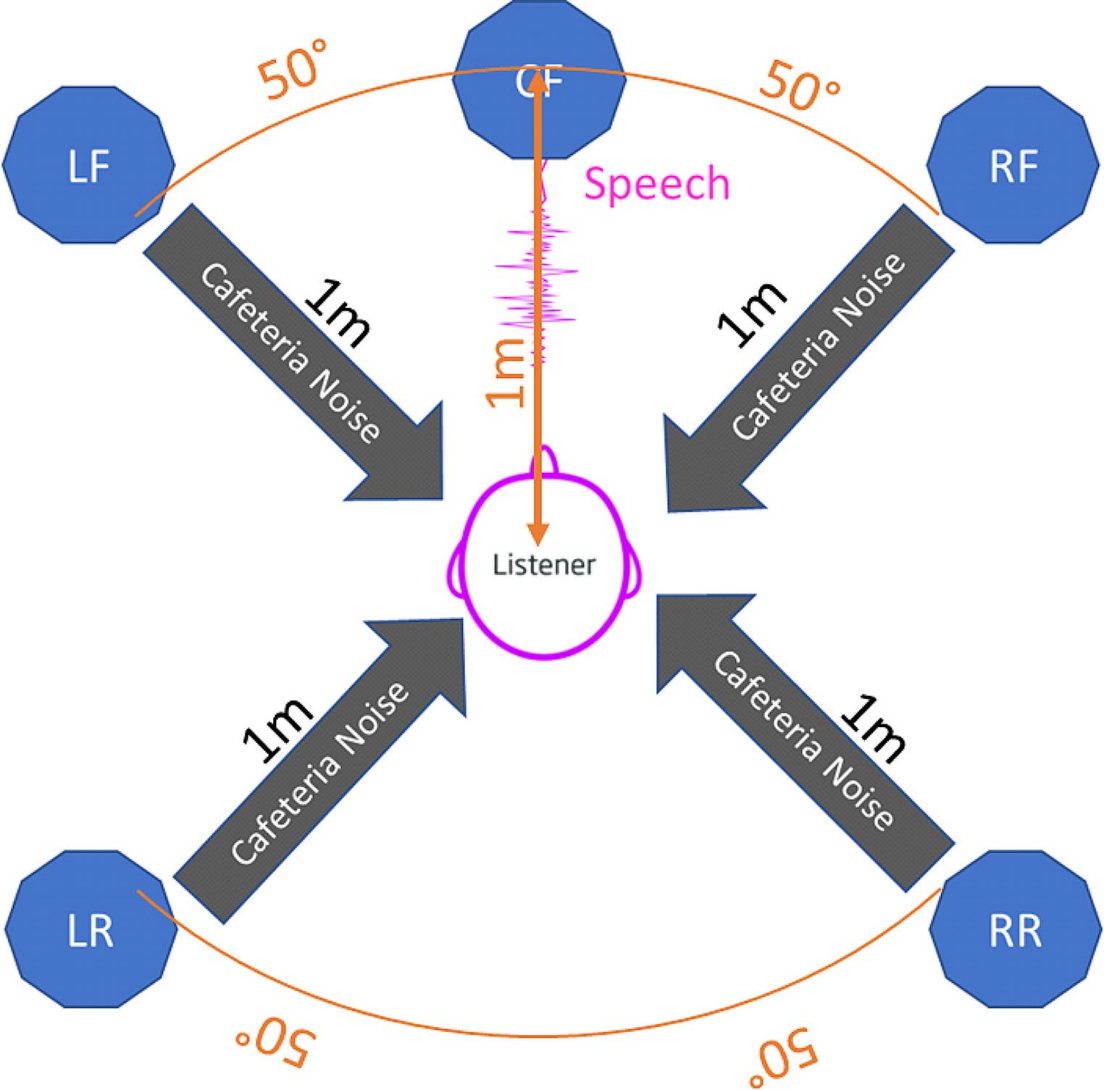
Speaker setup for the OLSA testing. Speech was presented from the centre speaker (CF) and the cafeteria noise from the surrounding speakers (LF, RF, LR, RR)

The test set up is shown in Fig. 2. Speech was presented from a GENELEC; 8020 A, active speaker and noise from 4 x KS digital; Coax C5, active speakers. All speakers were driven by a USB Soundcard: RME; FirefaceU.C. The cafeteria noise was presented as wave-files with the software: Steinberg; Cubase Elements, v9.5. The OLSA test was performed with the software: Oldenburger Messprogramme; v1.3 (1.3.9.0) research version. Calibration was checked prior each testing session using sound level meter (A-Weighting) NTI-Audio; XL2.

For the preferred setting, children were asked why they have chosen this particular setting and for the extreme setting whether they would ever choose this setting voluntarily.

### Subjective assessment

At the end of the first in-clinic appointment, subjects were given two ‘Einschätzung von Hörsituationen im Alltag von Kindern’ (E-HAK) [11] questionnaires and instructed to complete one after one week and the second a week later, during the home trial. The E-HAK questionnaire asked each subject to use the app in five different situations (1.playing sports, 2.street, 3.restaurant/party, 4.public transport, 5.in another situation where they wanted to try the app) and compare that particular situation between the hearing experience with the HA in default mode and HA adjusted with the app. The ease of each situation was rated on a five-point scale, ranging from “mostly easy-1” to “mostly difficult-5”. Rating “no change-0” indicates no difference. Each participant, depending on their individual daily routines and activities, could freely choose the five situations. While one might have considered for the sports activities a soccer training the other considered playing outside or the horse riding instead. This was completed with hearing aid only, and with hearing aid plus the app adjustments.

After the home trial, subjects returned for the second in-clinic session where the speech intelligibility testing was repeated. The same settings for the preferred and extreme conditions were used. The order of test conditions was randomised. At the end of the study all participants completed a structured exit interview guided by the E-HAK results.

### Statistics

Scores from the two speech intelligibility sessions were analysed as separate data points. A Shapiro-Wilks-Test was used to test for normal distribution of the data. Median values and boxplots and a non-parametric RM-ANOVA (repeated measures analysis of variance) were used for comparing the group OLSA results for the different app modalities. Significance testing was performed using Durbin-Conover pairwise comparisons. Average ratings for the E-HAK-Questionnaire were compared between the two sessions using a paired samples T-Test. All statistical analyses were performed in Jamovi version 2.3. Significance was set at *p* < 0.05.

## Results

**Fig. 3:**
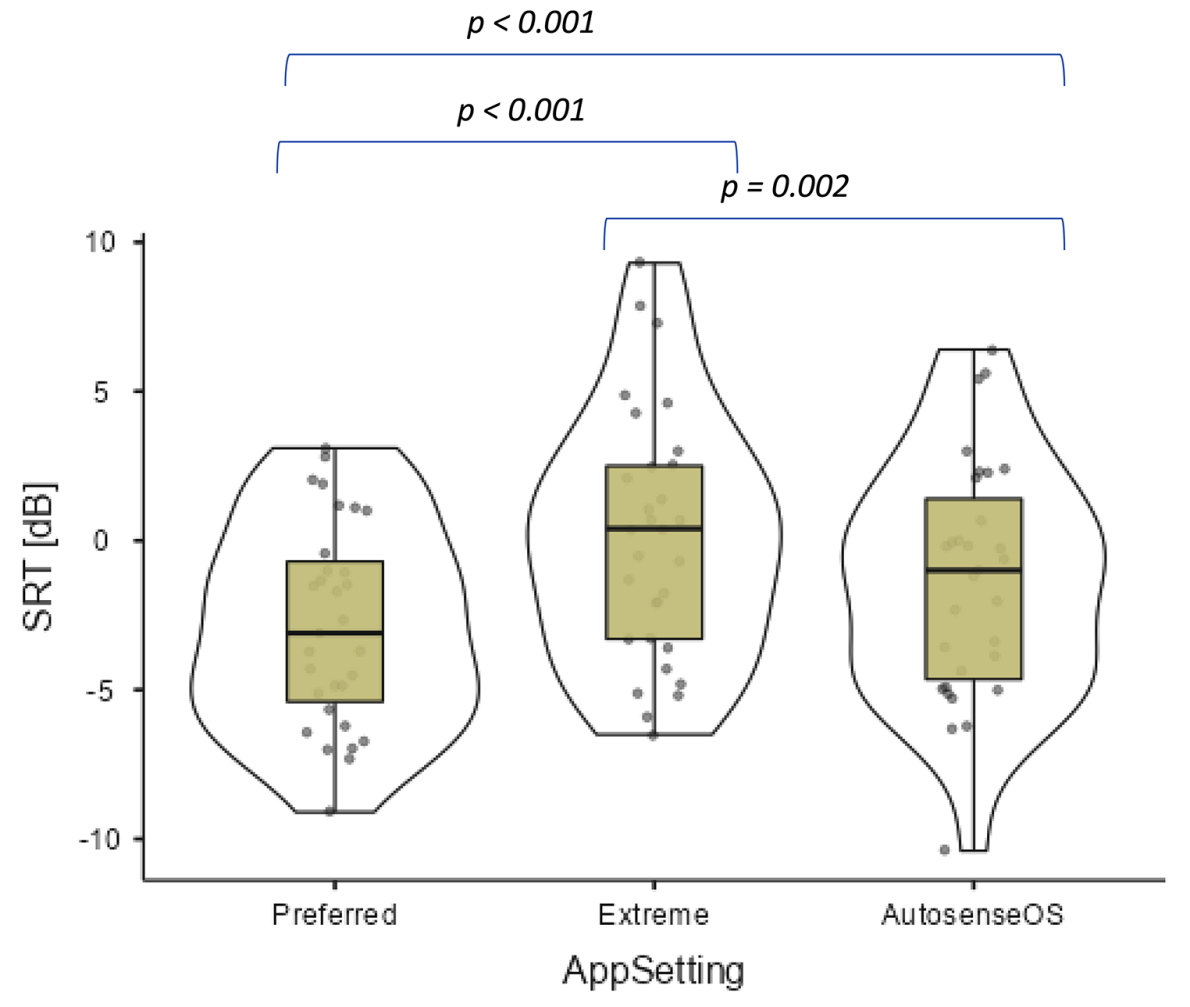
Boxplot of the combined OLSA-SRT result split by the Test condition. The solid line indicates median, box the interquartile range and whiskers the min-max range. The violin-shape outlies the distribution of the data. NB lower scores = better result

Speech reception threshold results for the three test conditions: AutoSense Sky OS, preferred and extreme, are shown in Table 1; Fig. 3. Scores from the two test sessions were included as individual data points. One child dropped out after the first face to face session was completed, and the analysis was based on 31 data points.

### Primary objective results

The preferred setting gave the best score compared to both Autosense Sky OS and the extreme setting. However, Autosense Sky OS was statistically better than the extreme setting. The gains in SRT of 2.1 and 3.5 dB for the preferred setting both represent a clinically meaningful change, which would be noticeable to the subject.

Descriptive statistics and results of the statistical testing are shown in the Table 1.

**Table 1:** Overview of the subject population, OLSA data and statistical significances. Number of data points is 31 from 16 subjects. Note: lower scores = better result

	SRT Autosense Sky OS	SRT Preferred	SRT Extreme	Preferred vs. Autosense Sky OS	Extreme vs. Autosense Sky OS	Preferred vs. extreme
Mean (SD)	-1.33 (3.93)	-2.83 (3.37)	0.08 (4.15)			
Median Range	-1.00 -10.4–6.40	-3.10 -9.10–3.10	0.40 -6.50–9.30			
SRT change p value				-2.1dB *p* < 0.001	1.4dB *p* = 0.002	-3.5dB *p* < 0.001
Shapiro-Wilk W	0.975	0.963	0.969			
Shapiro-Wilk p	0.656	0.340	0.496			

Four children aged 8–10 years old were included in the sample. To check if the performance of this younger group influenced the results of the whole group of subjects, a separate analysis was done for the 12 children the older group. The results remained the same with the Preferred better than Extreme (*p* < 0.001) and AutoSense Sky OS (*p* < 0.001) and AutoSense Sky OS better than Extreme (*p* = 0.012).

In the preferred test condition during the OLSA, changes made to Noise Reduction and Speech Focus were recorded.

### Secondary objective results

**Fig. 4:**
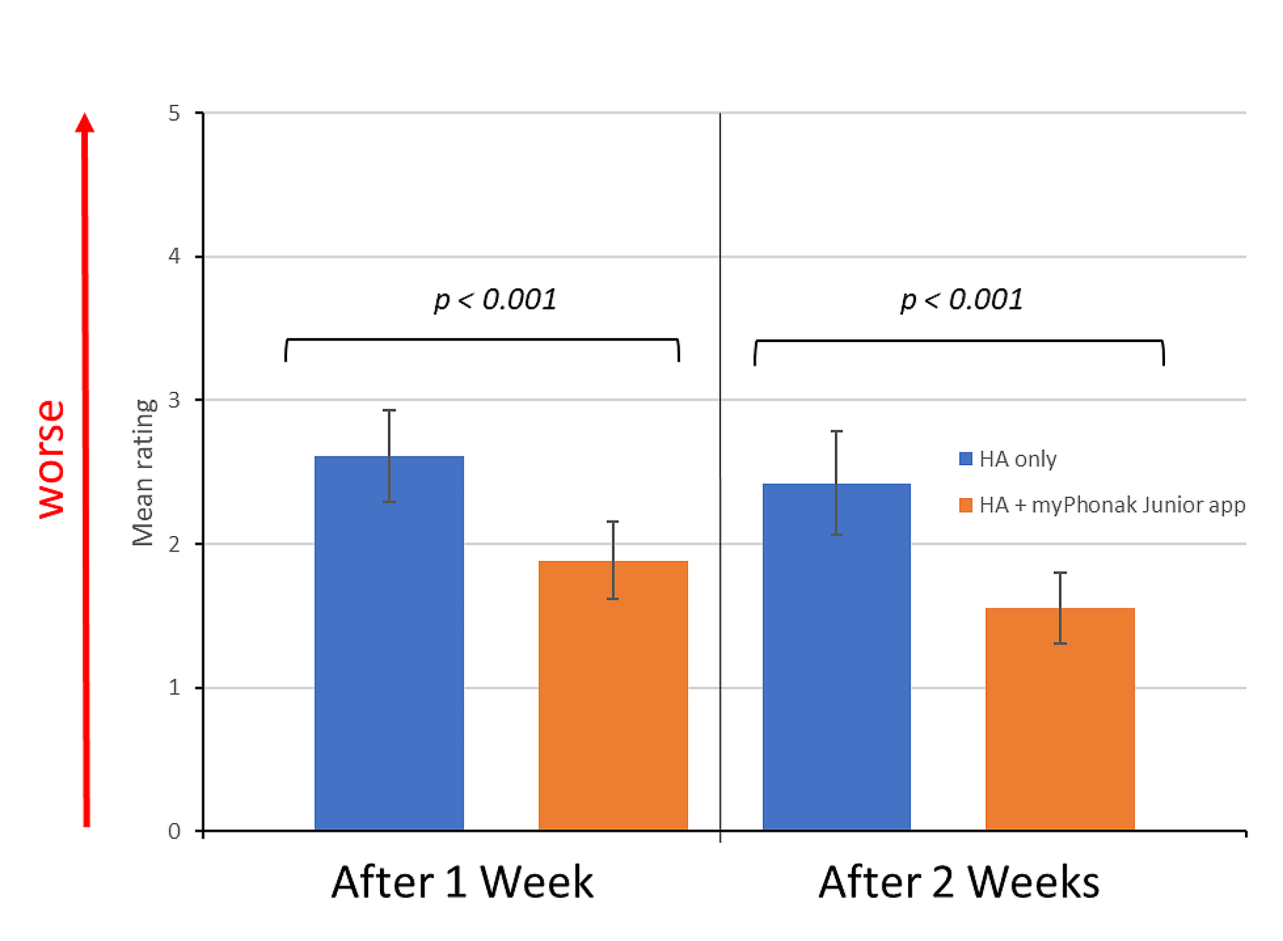
Average ratings of E-HAK-Questionnaire split by the time at completion and use of the app. Overall participants rated daily situations as being easier when the HA was adjusted with the app

The average score for the E-HAK-questionnaire is shown for each session in Fig. 4. The Shapiro-Wilks Test confirms a normal distribution of the ratings for each session and modality (HA-only vs. HA-the app). It was significantly easier to understand in a situation when adjusting the HA using the app than it was with the HA alone.

Results also showed that when the subjects were instructed to select extreme settings (max noise reduction, minimum speech focus) the SRT was significantly worse. Subjects were asked if they would use the hearing aid in this setting. None of the subjects said they would definitely use the extreme settings, but 30% reported “perhaps” they would use it depending on the situation. The subjects who said “perhaps” were in the older group. Of note, when the subjects were asked to create their own preferred settings, no one selected settings equivalent to the extreme settings.

### Exit interview results

The results from the interview conducted at the final study visit are of a descriptive nature and cannot be statistically analysed. However, we attempted to sum up and quantify similar responses in order to separate the very subjective comments to those raised by several participants. Two questions are quantified as an example reflecting the most frequent opinion or comment on the App.

Interview summary:

What was your experience with the app?

Good 4x = Age (14/12/17/15).

- very helpful when I was out with friends, when I was shopping, during sport activities like horse riding, table tennis, dancing.
- the children found it very good that they could do something for themselves and try to improve their situation individually according to their needs.

Sometimes good 2x = Age (12/10).

- helpful when I was out with friends, but unfortunately did not work properly, the connection to the HA was often interrupted, very unstable, that was annoying.

Good but poor connectivity 4x = Age (8/9/14/15).

- 8 and 9 y old forget always how to use, 14 and 15 y old had problems with the connectivity although they could handle it very well.

Poor (13/14/1016/11).

- Connectivity was too unstable. Children can’t save the different configurations, prevent me from important things, the app or the HI should know what I want to hear without my intervention (children ask for AI).

Would you continue to use the app in the future?

Yes: 6x = Age (8/10/11/13/14/14).

- Helpful in noisy surroundings.
- Helpful when wearing caps in winter or wearing masks due to Corona.
- Can hear when friends whisper.

No: 6x = Age (16/14/10/14/13/11).

- Takes too long, laborious, often not useful, disturbing, annoying.
- People think I’m rude and “play” with my phone.

Can’t decide: 3x = age (8/9/14).

- (8/9) want to have a mobile, but forget how to use in daily life.
- (14) handling is too laborious for him.

*For the complete interview responses*,* please refer to the data collection form (excel sheet).*

## Discussion

All included children were happy and highly motivated to participate in the study. The median age of the study group was 12 years ranging from 8 to 17 years. Since only four of the sixteen children who participated were below the age of 11 years, the entire group was more representative of teenagers. The schools in Germany suggest not to introduce smart phones to children below the age of 12 years or to start only after the elementary school. To some extent this reflects the social and cultural approach, where you are unlikely to see younger children with their own smart phones at an earlier age. Therefore, the motivation of the younger children (8–11) to participate was somewhat higher because of the fact that they would be allowed to use a smart phone during the study. For the teenagers however, the main motivation was to test if the app could help them understanding better in different situations by adjusting their HAs individually. Although the results indicated that the SRT for the younger group of subjects did not significantly influence the overall results, this aspect must be considered when interpreting the data and the resulting conclusions on the usefulness of the app.

At the initial study visit, all participants were instructed in using the app and making the relevant adjustments. In turn, each study participant had to confirm that they understood by completing dedicated tasks e.g. opening the app, adjusting the Volume, the Speech Focus and the Noise Reduction.

The younger group (< 11 years) easily understood the ergonomic aspects of the app and how to interact with it. However, at the initial visit and before the initial testing they needed repeated explanation on the meaning of individual adjustments and their use. Also, during the trial period at home, they tended to forget what and how to adjust in various situations. In their everyday lives, it was often no longer clear to them how they could use the app. This was not an issue for the older children.

Although, all study participants had prior experience with one version of the adaptive German Sentence Test in Noise, either the OLKISA (younger children) or OLSA, the specific adaptation of the noise for this study was challenging for the majority of participants. The cafeteria noise material included several competing speakers together with cutlery noises and was presented continuously during and in-between the individual testing and adjusting of the app.

When comparing the SRT scores for different app adjustments, there was a significant improvement of 2.1 dB with the self-adjusted “preferred” app condition than with the AutoSense Sky OS setting. This is encouraging and indicates that both groups can fine tune the app and improve the speech understanding in noise further. This aspect underlines the potential benefit of the app.

However, when comparing the preferred or AutoSense Sky OS setting to the “extreme” adjustment, both were significantly better by 3.5dB with the preferred and 1.4dB with the AutoSense Sky OS setting respectively. This difference was lower in the older group of children, but it is still significant. This may be of concern indicating that any miss-adjustment or un-intentional adjustment of the app to the extreme values can lead to significant drop of speech understanding in noisy listening environment.

Speech intelligibility was degraded for the Extreme settings (maximum Noise Reduction, most open beamformer). However, children did not select these settings voluntarily, they only used them during the specific test. When the children were asked if they would ever use these settings 70% said ‘No!’, 30% said perhaps for some specific situations.

Nevertheless, the risk emphasizes the need to make sure that only children and teenagers who have fully understood the purpose and the use of the app have access to it. Younger children and those not understanding the purpose of the app have a higher risk of misadjusting the settings and hearing worse in the noise than with the AutoSense Sky OS.

The questionnaire (E-HAK), used in the study, first provides information about the children’s activities at school (break times) and in their free time and home environment. It is important to consider that the study took place during the Corona pandemic (2021–2022) and children’s activities were restricted by this aspect.

In general, the group rated the listening in different daily situations as easier when adjusting the HA with the app than with HA alone without adjustments. We assume this is because they felt of having some control over the listening situation through the app but some might have indeed been able to improve the speech understanding, which we could confirm with the testing in noise. However, some felt uncomfortable in adjusting the app during a conversation and felt it was impolite towards the conversation partner to focus on the smartphone.

The younger participants gave a high rating on the usefulness of the app. However, based on parents’ feedback it was not possible to successfully integrate the use of the app into the daily routine. Here the likely driving factor for a good rating for the app adjustments of the HA was the wish to keep the smart phone after the study. All of the older children did already possess a smart phone and the possibility to control their HA was the main motivating aspect to participate.

Based on the exit interview-feedback, it appears that the younger and older children were all very motivated and curious to try the app in the structured study scenario and at home. The younger children, 8–11 years old, have shown that they could quickly understand how the app works and could also quickly master the use of a smart phone, even if they do not yet own one. However, they needed a lot of support from parents when using the app in daily situations, because they tended to forget how and why to use it. According to the parents they mostly just played with it. Therefore, we think it is difficult to control a meaningful use of the app to those below 11 years and do not see the app suitable for this particular group.

The older group was able to use the app in a more structured and focused manner and in some instances, they could improve the speech understanding in certain situation, which they were very pleased about. However, few children felt stressed by time and effort it took to fine-tune the app for the variety of daily situations, which sometimes change rapidly, despite the improvements they could achieve. Here the Autosense Sky OS feature is a good basis to dynamically adjust the level of noise reduction and beamforming based on the present listening environment without any need for manual adjustments. For a future revision of the app it should be considered to include the possibility to save custom settings in the app for simplifying the adjustments to a particular situation by just one button click.

## Conclusion

Based on the subjective and objective results from the study we do not see the app suitable for younger children below 11 years who have no prior smart phone experience and who may not be able to understand what changes they are making with the app to their HA. Here is potentially a higher risk for worsening speech understanding and a drop of performance if the HA is unintentionally adjusted in the extreme setting.

Overall, we think that the app is an easy-to-use way of controlling the level of Noise Reduction and BF in an intuitive and straight forward way for children 11 years and older. Further it has a potential to help teenagers customizing their HA in daily situations and improve the hearing in noise also beyond the already strong dynamic features such as Autosense Sky OS.
